# Early season co-circulation of influenza A(H3N2) and B(Yamagata): interim estimates of 2017/18 vaccine effectiveness, Canada, January 2018

**DOI:** 10.2807/1560-7917.ES.2018.23.5.18-00035

**Published:** 2018-02-01

**Authors:** Danuta M Skowronski, Catharine Chambers, Gaston De Serres, James A Dickinson, Anne-Luise Winter, Rebecca Hickman, Tracy Chan, Agatha N Jassem, Steven J Drews, Hugues Charest, Jonathan B Gubbay, Nathalie Bastien, Yan Li, Mel Krajden

**Affiliations:** 1 British Columbia Centre for Disease Control, Vancouver, Canada; 2 University of British Columbia, Vancouver, Canada; 3 Institut National de Santé Publique du Québec, Québec, Canada; 4 Laval University, Quebec, Canada; 5 Centre Hospitalier Universitaire de Québec, Québec, Canada; 6 University of Calgary, Calgary, Canada; 7 Public Health Ontario, Toronto, Canada; 8 Alberta Provincial Laboratory, Edmonton, Canada; 9 University of Alberta, Edmonton, Canada; 10 University of Toronto, Toronto, Canada; 11 National Microbiology Laboratory, Public Health Agency of Canada, Winnipeg, Canada

**Keywords:** Influenza, influenza virus, vaccine-preventable diseases, vaccines and immunisation, vaccine effectiveness, mid-season, genomics

## Abstract

Using a test-negative design, we assessed interim vaccine effectiveness (VE) for the 2017/18 epidemic of co-circulating influenza A(H3N2) and B(Yamagata) viruses. Adjusted VE for influenza A(H3N2), driven by a predominant subgroup of clade 3C.2a viruses with T131K + R142K + R261Q substitutions, was low at 17% (95% confidence interval (CI): −14 to 40). Adjusted VE for influenza B was higher at 55% (95% CI: 38 to 68) despite prominent use of trivalent vaccine containing lineage-mismatched influenza B(Victoria) antigen, suggesting cross-lineage protection.

The 2017/18 influenza season in Canada has been characterised by co-circulation of influenza A(H3N2) and B(Yamagata) viruses, the latter unusual so early in the season [1]. Most European countries are also experiencing simultaneous influenza A and B epidemics, with B(Yamagata) predominating [2], whereas the United States (US) has experienced a substantial epidemic due predominantly to influenza A(H3N2) [3].

The 2017/18 trivalent influenza vaccine (TIV) includes influenza A/Hong Kong/4801/2014(H3N2)-like (clade 3C.2a) and B/Brisbane/60/2008(Victoria-lineage)-like (clade 1A) antigens. The quadrivalent influenza vaccine (QIV) contains an additional influenza B/Phuket/3073/2013(Yamagata-lineage)-like (clade 3) antigen. The same components were included in the 2016/17 northern and 2017 southern hemisphere vaccines [4].

Low vaccine effectiveness (VE) for the 2017/18 season has been anticipated following the interim report from Australia indicating VE of just 10% during its 2017 influenza A(H3N2) epidemic [5]. In the context of exclusive QIV use, Australia reported higher VE of 57% against co-circulating influenza B viruses [5]. Here we report interim 2017/18 VE estimates for influenza A(H3N2) and influenza B from participating provinces of the Canadian Sentinel Practitioner Surveillance Network (SPSN), where QIV comprised less than one third of vaccine doses distributed overall through the publicly funded campaign.

## Vaccine effectiveness evaluation

VE was derived using a test-negative design [6-9]. Nasal/nasopharyngeal specimens and epidemiological data were collected from patients presenting within 7 days of onset of influenza-like illness (ILI) to community-based sentinel practitioners in Alberta, British Columbia, Ontario and Quebec. ILI was defined as acute onset of fever and cough and at least one other symptom including sore throat, myalgia, arthralgia or prostration. Fever was not a requirement for elderly adults 65 years of age and older. Vaccination status was based on patient and/or practitioner reporting of 2017/18 vaccination at least 2 weeks before symptom onset; patients vaccinated less than 2 weeks before onset or with unknown vaccination status/timing were excluded. Institutional review boards in each province provided ethical approval for the study.

Specimens collected from week 45 (starting 5 November 2017) to week 3 (ending 20 January 2018) were tested for influenza type/subtype by real-time RT-PCR at provincial public health reference laboratories. Sanger sequencing of the viral haemagglutinin gene was undertaken on a subset of original patient specimens collected up to 13 January 2018 to assess the contribution of genetic clades to VE estimates.

Odds ratios (OR) comparing test-positivity for influenza A(H3N2) or B between vaccinated and unvaccinated participants who were at least 1-year-old were calculated using logistic regression, adjusted for relevant covariates. VE was derived as (1 − OR) × 100%.

## Virological findings

**Figure 1:**
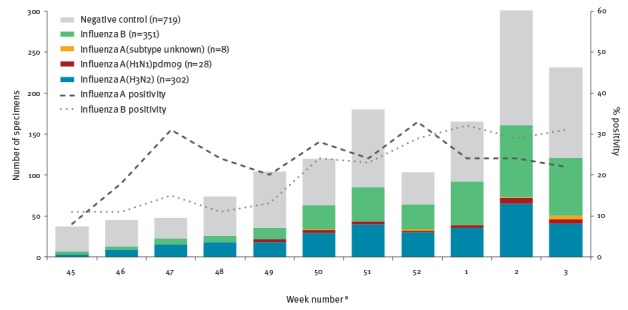
Influenza detections among eligible patients presenting with influenza-like illness by week of specimen collection, Canadian Sentinel Practitioner Surveillance Network, 5 November 2017–20 January 2018 (n = 1,408)

^a^ Based on week of specimen collection. Missing specimen collection dates were imputed as the laboratory accession date minus 2 days, the average time between specimen collection and accession dates among specimens with complete information for both values. Data for week 3 may be incomplete given delays in specimen processing and laboratory testing.

Among 1,408 eligible specimens, 689 (49%) tested positive for influenza, including 338 (49%) influenza A and 351 (51%) influenza B (Figure 1). Among the 330 (98%) subtyped influenza A viruses, 302 (92%) were A(H3N2) and 28 (8%) were A(H1N1)pdm09.

**Figure 2:**
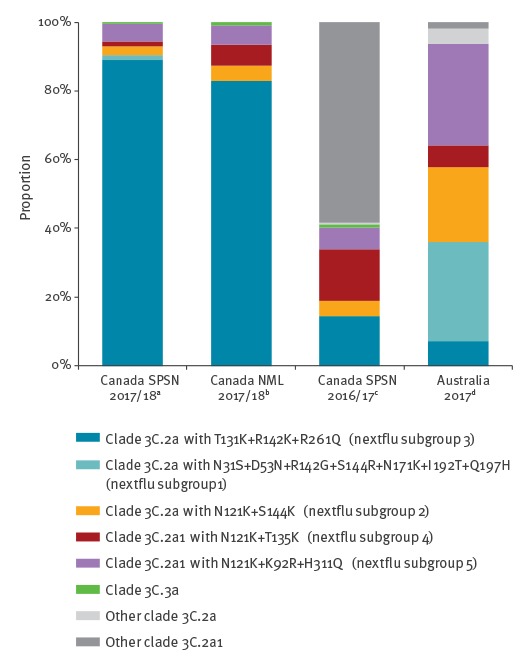
Clade distribution of influenza A(H3N2) variants, Canada, 2017/18 interim vaccine effectiveness evaluation vs other sources of data

NML: National Microbiology Laboratory; SPSN: Sentinel Practitioner Surveillance Network.

^a^ Sequencing of the haemagglutinin gene was attempted on a subset of available influenza-positive original patient specimens from the Canadian SPSN contributing to interim 2017/18 vaccine effectiveness evaluation. Sequencing was successful for 229 of 236 (97%) influenza A(H3N2) specimens attempted (collection dates: 5 November 2017 to 13 January 2018).

^b^ Sequencing based on 228 cultured isolates from inpatient and outpatient specimens submitted to Canada’s NML by provincial public health laboratories (Alberta, Saskatchewan, Manitoba, Ontario, Quebec, Nunavut) as at 26 January 2018 (collection dates: 1 November 2017 to 10 January 2018). Sequences were publicly available from the Global Initiative on Sharing All Influenza Data (GISAID) as acknowledged in Supplement 1. Of these 228 A(H3N2) viruses, 199 (87%) belonged to clade 3C.2a, and 189 (83%) overall belonged to the clade 3C.2a subgroup bearing T131K + R142K + R261Q substitutions (nextflu subgroup 3).

^c^ Sequencing based on 221 Canadian SPSN specimens included in the 2016/17 interim VE analysis (collection dates: 1 November 2016 to 16 January 2017) [9]. Among Canadian 2016/17 A(H3N2) viruses, 43 (19%) belonged to clade 3C.2a and 32 (14%) of A(H3N2) viruses overall belonged to the clade 3C.2a subgroup bearing T131K + R142K + R261Q substitutions (nextflu subgroup 3).

^d^ Sequencing based on 206 Australian specimens included in the 2017 interim VE analysis (collection dates: 1 May 2017 to 24 September 2017) [5]. Among Australian 2017 A(H3N2) viruses, 128 (62%) belonged to clade 3C.2a and 15 (7%) of A(H3N2) viruses overall belonged to the clade 3C.2a subgroup bearing T131K + R142K + R261Q substitutions (nextflu subgroup 3).

Most sequenced influenza A(H3N2) viruses belonged to genetic clade 3C.2a (213/229; 93%) and of these most (204/213; 96%) belonged to a single genetic subgroup of 3C.2a (denoted subgroup 3 by nextflu.org [10]), bearing antigenic site A substitutions T131K and R142K and antigenic site E substitution R261Q (Table 1). Overall 89% of influenza A(H3N2) viruses belonged to clade 3C.2a subgroup 3, which is similar to other surveillance observations from Canada (83%) (Figure 2) and to recent reports from Europe [11]. However, this profile for the 2017/18 season is different from that found by the Canadian SPSN during 2016/17 or by Australia during its 2017 epidemic, when a greater mix of genetic variants contributed to interim analyses and only 14% and 7%, respectively, of influenza A(H3N2) viruses belonged to subgroup 3 (Figure 2). 

**Table 1:** Virological profile of influenza specimens contributing to interim 2017/18 vaccine effectiveness evaluation based on Sanger sequencing, Canadian Sentinel Practitioner Surveillance Network, 5 November 2017–13 January 2018 (n = 462)

Genetic clade with substitutions (nextflu subgroup)^a^	Alberta		British Columbia		Ontario		Quebec		Overall	
	n	%	n	%	n	%	n	%	n	%
Influenza A(H3N2)	114	100	38	100	50	100	27	100	229	100
**Clade 3C.2a**	**105**	**92**	**36**	**95**	**48**	**96**	**24**	**89**	**213**	**93**
+ N31S + D53N + R142G + S144R + N171K + I192T + Q197H (subgroup 1)	2	2	0	0	0	0	1	4	3	1
+ N121K + S144K (subgroup 2)^b^	1	1	1	3	3	6	1	4	6	3
+ T131K + R142K + R261Q (subgroup 3)^c^	102	89	35	92	45	90	22	81	204	89
**Clade 3C.2a1**	**9**	**8**	**2**	**5**	**1**	**2**	**3**	**11**	**15**	**7**
+ N121K + T135K (subgroup 4)^d^	2	2	1	3	0	0	0	0	3	1
+ N121K + K92R + H311Q (subgroup 5)^e^	7	6	1	3	1	2	3	11	12	5
**Clade 3C.3a**	**0**	**0**	**0**	**0**	**1**	**2**	**0**	**0**	**1**	**0**
Influenza B	76	100	83	100	63	100	11	100	233	100
Yamagata lineage clade 3^f^	76	100	82	99	62	98	7	64	227	97
Victoria lineage clade 1A^g^	0	0	1	1	1	2	4	36	6	33

Sequencing of the haemagglutinin gene was attempted on a subset of available influenza-positive original patient specimens contributing to interim 2017/18 vaccine effectiveness evaluation. Sequencing was successful for 229 of 236 (97%) influenza A(H3N2) specimens and 233 of 246 (95%) influenza B specimens (collection dates: 5 November 2017 to 13 January 2018).

^a^ Subgroup name as assigned by nextflu.org [10].

^b^ Five of six viruses in this subgroup had additional substitutions T135K (associated with loss of a potential glycosylation site) + R150K + R261Q; the sixth virus had N122D (associated with loss of a potential glycosylation site) + N171D + S262N.

^c^ 44 of 204 (22%) viruses in this subgroup had an additional substitution K92R and 28 of 204 (14%) had A212T.

^d^ All viruses in this subgroup had additional substitutions G78D + Y94H + V182I; two viruses also had N122D (associated with loss of a potential glycosylation site).

^e^ All but two viruses in this subgroup had additional substitutions T135K (associated with loss of a potential glycosylation site) + E62G + R142G; one virus also had N122D (associated with loss of a potential glycosylation site). One of the other two viruses had T135N.

^f^ All but one of the viruses in this subgroup had additional substitutions L172Q + M251V; one virus had M251V without L172Q.

^g^ Five of six viruses in this subgroup had a deletion at position 162–163.

Virtually all sequenced influenza B viruses were B(Yamagata) clade 3 (227/233; 97%) and all but one had L172Q + M251V non-antigenic site substitutions, the dominant genetic variant circulating globally since 2015 [11]; one virus had M251V without L172Q. Six viruses were influenza B(Victoria) clade 1A (five with a deletion at position 162–163) [11].

## Epidemiological findings

Most (64%) participants were adults 20–64-years-old. More influenza B cases (20%) than controls (11%) were children 9–19-years-old (p < 0.01) (Table 2). More cases of influenza A(H3N2) (25%; p = 0.07) and influenza B (27%; p < 0.01) were 50–64-years-old compared with controls (18%).

**Table 2:** Participant profile, interim 2017/18 influenza vaccine effectiveness evaluation, Canadian Sentinel Practitioner Surveillance Network, 5 November 2017–20 January 2018 (n = 1,408)

Characteristic	All participants (column %)								% vaccinated^a^ (row %)								
	Influenza A(H3N2) cases		p value^b^	Influenza B cases		p value^c^	Negative controls		Influenza A(H3N2) cases		p value^d^	Influenza B cases		p value^d^	Negative controls		p value^d^
	n	%		n	%		n	%	n	%		n	%		n	%	
Overall	302	100	NA	351	100	NA	719	100	100	33	NA	80	23	NA	253	35	NA
Age group (years)																	
1–8	18	6	0.07	21	6	< 0.01	64	9	2	11	< 0.01	0	0	< 0.01	10	16	< 0.01
9–19	31	10		70	20		82	11	7	23		1	1		15	18	
20–49	126	42		117	33		325	45	34	27		21	18		91	28	
50–64	77	25		95	27		131	18	26	34		30	32		48	37	
≥ 65	50	17		48	14		117	16	31	62		28	58		89	76	
Median (range)	43	(2–87)	0.17	43	(1–91)	0.53	39	(1–96)	53.5	(3–87)	< 0.01	61.5	(12–91)	< 0.01	52	(1–96)	< 0.01
Sex																	
Female	185	62	0.45	205	59	0.95	421	59	71	38	0.02	55	27	0.03	162	38	0.04
Male	115	38		143	41		291	41	29	25		24	17		90	31	
Unknown	2	NA	NA	3	NA	NA	7	NA	0	NA	NA	1	NA	NA	1	NA	NA
Co-morbidity^e^																	
No	226	77	0.57	262	80	0.12	524	76	63	28	< 0.01	46	18	< 0.01	155	30	< 0.01
Yes	66	23		65	20		168	24	33	50		31	48		92	55	
Unknown	10	NA	NA	24	NA	NA	27	NA	4	NA	NA	3	NA	NA	6	NA	NA
Province																	
Alberta	127	42	< 0.01	91	26	0.10	201	28	40	31	0.10	14	15	< 0.01	75	37	< 0.01
British Columbia	48	16		107	30		200	28	16	33		31	29		70	35	
Ontario	77	25		114	32		203	28	33	43		33	29		84	41	
Quebec	50	17		39	11		115	16	11	22		2	5		24	21	
Specimen collection interval from ILI onset (days)^f^																	
≤ 4	239	79	< 0.01	252	72	0.42	499	69	78	33	0.73	58	23	0.87	170	34	0.34
5–7	63	21		99	28		220	31	22	35		22	22		83	38	
Median (range)	3	(0–7)	< 0.01	3	(0–7)	0.85	3	(0–7)	3	(0–7)	0.18	3	(1–7)	0.96	3	(0–7)	0.88
Specimen collection month																	
November	38	13	0.10	23	7	< 0.01	129	18	6	16	0.04	1	4	0.03	27	21	< 0.01
December	124	41		117	33		269	37	47	38		23	20		99	37	
January	140	46		211	60		321	45	47	34		56	27		127	40	
2017/18 vaccination status																	
Vaccination without regard to timing^g^	112/ 314	36	0.48	87/ 358	24	< 0.01	285/ 751	38	NA	NA	NA	NA	NA	NA	NA	NA	NA
≥ 2 weeks before ILI onset	100	33	0.52	80	23	< 0.01	253	35	NA	NA	NA	NA	NA	NA	NA	NA	NA

ILI: influenza-like illness; NA: not applicable.

The number of participants with unknown sex or comorbidity are shown in table but excluded from the denominator for calculating percentages.

^a^ Vaccination status based on patient and/or practitioner report; defined as receipt of 2017/18 seasonal influenza vaccine ≥ 2 weeks before symptom onset. Patients vaccinated < 2 weeks before onset or with unknown vaccination status/timing were excluded.

^b^ p value for comparison of influenza A(H3N2) cases to negative controls. Differences were compared using the chi-squared test or Wilcoxon rank-sum test.

^c^ p value for comparison of influenza B cases to negative controls. Differences were compared using the chi-squared test or Wilcoxon rank-sum test.

^d^ p value for comparison of vaccinated participants to unvaccinated participants. Differences were compared using the chi-squared test or Wilcoxon rank-sum test.

^e^ Includes chronic co-morbidities that place individuals at higher risk of serious complications from influenza as defined by Canada’s National Advisory Committee on Immunization (NACI), including: heart, pulmonary (including asthma), renal, metabolic (such as diabetes), blood, cancer or immunocompromising conditions, conditions that compromise management of respiratory secretions and increase risk of aspiration, or morbid obesity (body mass index ≥ 40).

^f^ Missing specimen collection dates were imputed as the laboratory accession date minus 2 days, the average time between specimen collection and accession dates among specimens with complete information for both values.

^g^ Participants who received seasonal 2017/18 influenza vaccine < 2 weeks before ILI onset or for whom vaccination timing was unknown were excluded from the primary analysis. They are included here for assessing vaccination regardless of timing for comparison to other sources of vaccination coverage.

Adjusted VE against influenza A(H3N2) was 17% (95% confidence interval (CI): −14 to 40) overall and 10% (95% CI: −31 to 39) in adults 20–64-years-old (Table 3). The corresponding VE against influenza B was higher at 55% (95% CI: 38 to 68) and 40% (95% CI: 10 to 60), respectively. With adjustment by calendar month (rather than 2-week interval) and the same covariates otherwise, VE with restriction to influenza B viruses of known Yamagata lineage (239/351; 68%) was 58% (95% CI: 38 to 71) overall and 47% (95% CI: 16 to 67) in adults 20–64-years-old. Adjusted VE against any influenza A and B combined was 42% (95% CI: 25 to 55) overall and 31% (95% CI: 6 to 49) in adults 20–64-years-old.

**Table 3:** Interim 2017/18 vaccine effectiveness estimates, Canadian Sentinel Practitioner Surveillance Network, 5 November 2017–20 January 2018 (n = 1,408)

Model	Influenza A(H3N2)		Influenza B		Overall (A and B)	
All participants						
Sample size	n vac / N	% vac	n vac / N	% vac	n vac / N	% vac
Cases	100/302	33	80/351	23	186/689	27
Controls	253/719	35	253/719	35	253/719	35
Vaccine effectiveness	VE %	95% CI	VE %	95% CI	VE %	95% CI
Unadjusted	9	−21 to 31	46	27 to 59	32	14 to 46
Age group	15	−15 to 38	49	30 to 63	36	18 to 50
Province	8	−23 to 31	49	31 to 62	34	16 to 47
Specimen collection interval	8	−23 to 31	46	27 to 59	31	14 to 45
Calendar time	13	−16 to 35	52	35 to 64	38	21 to 51
Full covariate adjustment^a^	17	−14 to 40	55	38 to 68	42	25 to 55
Participants 20–64 years-old						
Sample size	n vac / N	% vac	n vac / N	% vac	n vac / N	% vac
Cases	60/203	30	51/212	24	113/439	26
Controls	139/456	30	139/456	30	139/456	30
Vaccine effectiveness	VE %	95% CI	VE %	95% CI	VE %	95% CI
Unadjusted	4	−37 to 33	28	−5 to 50	21	−6 to 41
Full covariate adjustment^a^	10	−31 to 39	40	10 to 60	31	6 to 49

CI: confidence interval; n vac: number vaccinated; N: number total; % vac: percentage vaccinated; VE: vaccine effectiveness.

^a^ Analyses adjusted for age group (categorical: 1–8, 9–19, 20–49, 50–64 or ≥ 65 years), province (categorical: Alberta, British Columbia, Ontario or Quebec), specimen collection interval (categorical: ≤ 4 or 5–7 days) and calendar time (categorical: 2-week intervals based on week of specimen collection).

## Discussion

In most other interim analyses by the Canadian SPSN, type B viruses comprised less than 10% of influenza detections, whereas in 2017/18, they were identified in an equal proportion with influenza A(H3N2) [7-9]. Although the reasons for an earlier influenza B onset are unclear, Canada experienced a substantial influenza A(H3N2) epidemic in 2016/17 that may have altered population immunity and the overall 2017/18 influenza A(H3N2) contribution [9].

Nearly all (93%) characterised influenza A(H3N2) viruses were clade 3C.2a, a change from 2016/17 when most (80%) of the A(H3N2) viruses instead belonged to clade 3C.2a1 [9]. Furthermore, a single subgroup of clade 3C.2a with T131K + R142K + R261Q substitutions (i.e. nextflu subgroup 3 [10]) is currently predominating (89% of influenza A(H3N2) viruses), whereas a more heterogeneous mix of genetic variants contributed in Canada during 2016/17 [9] and in Australia during their 2017 epidemic [5]. Changes in the proportionate contribution and emerging predominance of clade 3C.2a variants among circulating influenza A(H3N2) viruses are important to monitor globally. The World Health Organization will decide in February 2018 whether to update the current clade 3C.2a vaccine antigen for the 2018/19 northern hemisphere vaccine, having already chosen a clade 3C.2a1 strain for the southern hemisphere’s 2018 vaccine [4].

Our 2017/18 interim VE estimate of 17% (95% CI: −14 to 40) is less than half that reported for the same A(H3N2) vaccine in 2016/17, including interim analyses by the Canadian SPSN (42%; 95% CI: 18 to 59) [9], the US Flu VE Network (43%; 95% CI: 29 to 54) [12] and the European I-MOVE Network (38%; 95% CI: 21 to 51) [13]. Our estimate is also lower than end-of-season estimates from Canada (37%; 95% CI: 20 to 51) [14] and the US (34%; 95% CI: 24 to 42) for 2016/17 [15], and lower than is expected generally for influenza A(H3N2) vaccines (33%; 95% CI: 26 to 39) [16].

Our 2017/18 interim VE for influenza A(H3N2) is more comparable to the 2017 southern hemisphere interim VE of 10% (95% CI: −16 to 31) reported from Australia [5]. Differences in virological and participant profiles, as well as the stage of the epidemic, have to be taken into account when comparing VE estimates across studies. Working-age adults comprised the majority of participants in both studies and the 2017/18 interim VE against influenza A(H3N2) among Canadian SPSN participants 20–64-years-old (10%; 95% CI: −31 to 39) is also comparable to the 2017 estimate reported from Australia for 15–64-year-olds (16%; 95% CI: −11 to 36). Sample size for other age groups (e.g. children, elderly adults) was too limited to derive reliable interim estimates or to inform protection in specific high-risk groups.

All influenza vaccine manufacturing in Canada is egg-based. Mutations that arise from egg adaptation of the vaccine strain may affect VE, an issue also identified for the current season’s A(H3N2) vaccine component [17,18]. In Canada this season, antigenic characterisation of influenza A(H3N2) viruses has only been presented in relation to a cell-propagated version of the vaccine reference strain; characterisation against an egg-based version has not been reported [1]. Among the small subset of Canadian viruses that could be successfully characterised, all were considered antigenically similar to the cell-propagated vaccine strain [1]. Conversely, where relatedness to the egg-propagated version of the vaccine strain has been specifically explored elsewhere, more variability has been identified, with a greater proportion of viruses considered antigenically distinct from the egg-propagated version [5,11,19]. 

We found higher VE of 55% (95% CI: 38 to 68) against influenza B despite prominent use of TIV containing a B(Victoria) antigen that was lineage-mismatched to almost exclusively B(Yamagata) viruses. Approximately 70% of vaccine doses distributed in SPSN provinces during the 2017/18 season were TIV, albeit with regional variation that will be explored in end-of-season analyses. Substantial cross-lineage VE for influenza B has been observed previously [20], including during the prior 2016/17 season in Canada when VE against lineage-mismatched influenza B using the same B(Victoria) TIV component was 73% (95% CI: 52 to 84) [14] and QIV comprised an even smaller proportion (< 25%) of vaccine doses distributed. Our estimate for the current season is comparable to the interim VE of 57% (95% CI: 41 to 69) for influenza B reported from Australia, despite exclusive use of QIV in that country [5].

Other agent–host and immuno–epidemiological interactions, including birth cohort effects induced by differential prime–boost exposures, may also play a role in VE [21]. The effect of prior vaccination history was not assessed here owing to sample size limitations, but will be explored as part of the end-of-season analyses.

## Conclusions

As reported from Australia for the 2017 southern hemisphere vaccine, interim estimates from Canada for the 2017/18 northern hemisphere vaccine indicate low VE of less than 20% against influenza A(H3N2), notably among working-age adults. While the influenza A(H3N2) epidemic continues, adjunct protective measures should be reinforced to minimise the associated disease burden in high-risk individuals [22]. Interim 2017/18 VE estimates against influenza B are higher at 55% despite prominent TIV use, suggesting cross-lineage protection.

## Genbank accession numbers (SPSN sequences)

Pending.
