# Addendum for Euro Surveill. 2018;23(5)

**DOI:** 10.2807/1560-7917.ES.2018.23.30.1807262

**Published:** 2018-07-26

**Authors:** Eurosurveillance editorial team

**Affiliations:** 1 European Centre for Disease Prevention and Control (ECDC), Stockholm, Sweden

In the article entitled ‘Early season co-circulation of influenza A(H3N2) and B(Yamagata): interim estimates of 2017/18 vaccine effectiveness, Canada, January 2018’ by D Skowronski et al., published on 1 February 2018, the GenBank accession numbers of the influenza virus sequences were added on 17 July 2018 at the request of the authors.

