# Authors’ correction for Euro Surveill. 2018;23(5)

**DOI:** 10.2807/1560-7917.ES.2018.23.30.1807261

**Published:** 2018-07-26

**Authors:** Eurosurveillance editorial team

**Affiliations:** 1 European Centre for Disease Prevention and Control (ECDC), Stockholm, Sweden

As requested by the authors of the article ‘Early season co-circulation of influenza A(H3N2) and B(Yamagata): interim estimates of 2017/18 vaccine effectiveness, Canada, January 2018’, published on 1 February 2018, a cell value was changed in Table 1, row label ‘Victoria lineage clade 1A’, column label ‘Overall %’, as it mistakenly stated ‘33%’ instead of ‘3%’. The mistake was corrected on 20 July 2018.

